# Dual Targeting of the p38 MAPK-HO-1 Axis and cIAP1/XIAP by Demethoxycurcumin Triggers Caspase-Mediated Apoptotic Cell Death in Oral Squamous Cell Carcinoma Cells

**DOI:** 10.3390/cancers12030703

**Published:** 2020-03-16

**Authors:** Ming-Hsien Chien, Wei-En Yang, Yi-Chieh Yang, Chia-Chi Ku, Wei-Jiunn Lee, Meng-Ying Tsai, Chiao-Wen Lin, Shun-Fa Yang

**Affiliations:** 1 Graduate Institute of Clinical Medicine, College of Medicine, Taipei Medical University, Taipei 11031 Taiwan; mhchien1976@gmail.com (M.-H.C.); rafiyang@tmu.edu.tw (Y.-C.Y.); laboyku@gmail.com (C.-C.K.); 2 TMU Research Center of Cancer Translational Medicine, Taipei Medical University, Taipei 11031, Taiwan; 3 Pulmonary Research Center, Wan Fang Hospital, Taipei Medical University, Taipei 11696, Taiwan; 4 Traditional Herbal Medicine Research Center, Taipei Medical University Hospital, Taipei 11031, Taiwan; 5 Institute of Medicine, Chung Shan Medical University, Taichung 40201, Taiwan; weienyang@gmail.com (W.-E.Y.); vickyfatfat5252@gmail.com (M.-Y.T.); 6 Department of Medical Research, Chung Shan Medical University Hospital, Taichung 40201, Taiwan; 7 Department of Medical Research, Tungs’ Taichung MetroHarbor Hospital, Taichung 433, Taiwan; 8 Department of Medical Education and Research, Wan Fang Hospital, Taipei Medical University, Taipei 11696, Taiwan; lwj5905@gmail.com; 9 Department of Urology, School of Medicine, College of Medicine, Taipei Medical University, Taipei 40201, Taiwan; 10 Graduate Institute of Oral Sciences, Chung Shan Medical University, Taichung 40201, Taiwan; 11 Department of Dentistry, Chung Shan Medical University Hospital, Taichung 40201, Taiwan

**Keywords:** demethoxycurcumin, apoptosis, inhibitor of apoptosis proteins, heme oxygenase-1, oral squamous cell carcinoma

## Abstract

Demethoxycurcumin (DMC) is a curcumin analogue with better stability and higher aqueous solubility than curcumin after oral ingestion and has the potential to treat diverse cancers, including oral squamous cell carcinoma (OSCC). The aim of this study was to investigate the anticancer effects and underlying mechanisms of DMC against OSCC. We found that DMC suppressed cell proliferation via simultaneously inducing G2/M-phase arrest and cell apoptosis. Mechanistic investigations found that the downregulation of cellular IAP 1 (cIAP1)/X-chromosome-linked IAP (XIAP) and upregulation of heme oxygenase-1 (HO-1) were critical for DMC-induced caspase-8/-9/-3 activation and apoptotic cell death. Moreover, p38 mitogen-activated protein kinase (MAPK) and c-Jun N-terminal kinase (JNK)1/2 were activated by DMC treatment in OSCC cells, and only the inhibition of p38 MAPK significantly abolished DMC-induced HO-1 expression and caspase-8/-9/-3 activation. The analyses of clinical datasets revealed that patients with head and neck cancers expressing high HO-1 and low cIAP1 had the most favorable prognoses. Furthermore, a combinatorial treatment of DMC with epidermal growth factor receptor (EGFR) tyrosine kinase inhibitor, gefitinib, significantly enhanced the inhibitory effect of gefitinib on the proliferation of OSCC cells. Overall, the current study supported a role for DCM as part of a therapeutic approach for OSCC through suppressing IAPs and activating the p38-HO-1 axis.

## 1. Introduction

Oral squamous cell carcinoma (OSCC) accounts for 90% of head and neck cancers located in the oral cavity and is the sixth leading cancer by incidence worldwide [1]. Despite the development of treatment modalities for OSCC such as surgical extraction, chemoradiotherapy, or epidermal growth factor receptor (EGFR)-targeting therapies in the last three decades, the prognosis of OSCC is still poor due to resistance to treatment modalities and cancer recurrence with a five-year survival rate of <50% [2]. Moreover, some of these drugs may also exhibit cytotoxic effects on normal cells, thus causing unpleasant side effects. Due to the unsatisfactory results of these standard treatments for OSCC, identifying new agents is crucial.

Solid malignant tumors, such as OSCC, have the potential for rapid and unlimited growth due to resistance to apoptosis [3]. Escape from apoptosis allows cancer cells to survive longer and accumulate mutations. The overexpression of a family of antiapoptotic proteins termed inhibitor of apoptosis (IAP) proteins, including cellular IAP 1 and 2 (cIAP1 and cIAP2, encoded by *BIRC2* and *BIRC3*), X-chromosome-linked IAP (XIAP, encoded by *BIRC4*), and survivin was reported to confer resistance to radiation therapy and chemotherapy and cause poor prognoses of patients with head and neck cancers, including OSCC [4,5,6,7]. Therefore, several preclinical and clinical trials aimed at reducing IAP expression were performed on head and neck cancers. For instance, studies demonstrated that LCL161, a cIAP1 antagonist, sensitizes a panel of OSCC cell lines to Fas ligand (FasL) treatment [8]. Moreover, the dual antagonist of cIAP/XIAP, ASTX660, was reported to induce the radiosensitization of head and neck cancers [9].

Heme oxygenase-1 (HO)-1 protein, in non-neoplastic cells, is encoded by a stress-inducible gene (*HMOX1*) but, as soon as it has been translated, the protein is active to degrade heme to biliverdin, carbon monoxide (CO), and free iron [10]. HO-1 was reported to be overexpressed or downregulated in different cancer types and has a multifaceted role in cancer development through regulating apoptosis, angiogenesis, and metastasis [11]. For example, the overexpression of HO-1 can enhance the proliferative or metastatic abilities of pancreatic cancer [12], melanomas [13], and rhabdomyosarcomas [14] in vitro and in vivo. In contrast, the overexpression of HO-1 exerts antiproliferative or anti-invasive abilities in breast, lung, and liver cancers [15,16,17]. In OSCC patients, HO-1 expression levels were shown to be negatively correlated with cervical lymph node metastasis [18], but the role of HO-1 in OSCC still requires elucidation.

Curcuminoids comprise three bioactive components, curcumin (CUR), demethoxycurcumin (DMC), and bisdemethoxycurcumin (BDMC), which are isolated from the rhizomes of *Curcuma longa* Linn [19]. CUR, the most abundant component of curcuminoids, was demonstrated to have anticancer potential due to its capacity to modulate apoptosis-related regulators including IAP or HO-1 in different cancer types [20,21]. However, previous reports have indicated that CUR is a poorly water-soluble compound especially in water at acidic or neutral pH and is unstable in alkaline or high-pH conditions. Therefore, the oral absorption of CUR is dramatically influenced by its low solubility, and the poor stability of CUR is observed in gastrointestinal fluids [22,23]. Due to the low oral bioavailability, the clinical use of CUR in cancer therapy is limited. Recently, accumulating evidence proved that the second most abundant active component of curcuminoids, DMC, is a more efficient and stable agent than CUR for cancer therapy [24,25,26]. Until now, the precise cellular mechanisms of DMC against OSCCs have not yet been fully clarified.

In this study, we investigated the anticancer effect of DMC against human primary and metastatic OSCC cell lines. In addition, we further explored whether the effect of DMC is related to IAP and HO-1 expressions.

## 2. Results

### 2.1. DMC Exerts Antiproliferative Activity and Causes G2/M Cell Cycle Arrest in OSCC Cells

**Figure 1:**
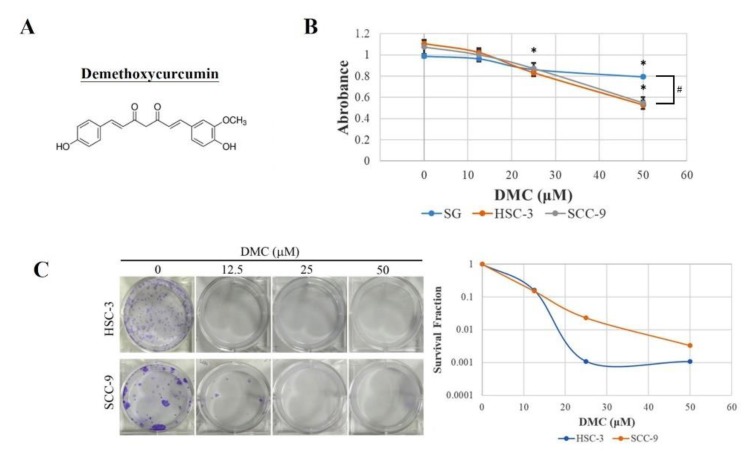

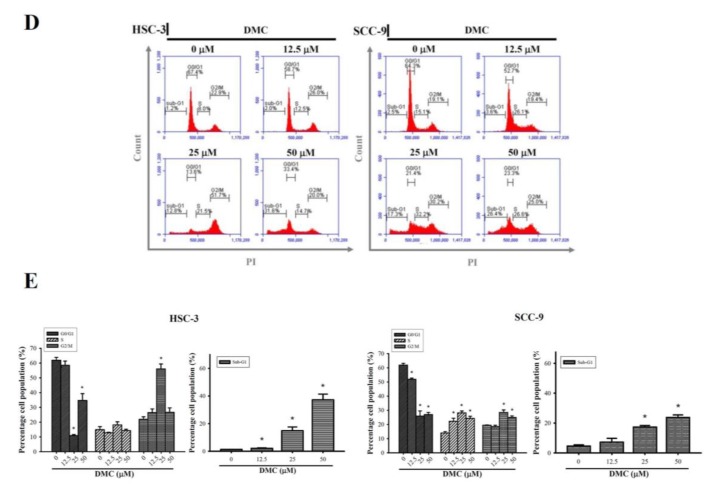
Demethoxycurcumin (DMC) inhibits the proliferation and colony formation via inducing G_2_/M phase arrest in oral squamous cell carcinoma (OSCC) cells. (**A**) The chemical structure of DMC. (**B**) Two OSCC cell lines, SCC-9 and HSC-3, and one normal gingival epithelial cell line, SG, were treated with indicated concentrations of DMC (12.5, 25, and 50 μM) or DMSO (vehicle control) for 24 h, and a thiazolyl blue tetrazolium bromide (MTT) assay was performed to determine the cell viability. * *p* < 0.05, compared to the DMSO-treated group. # *p* < 0.05, compared to the OSCC cells. (**C**) After 24 h treatment of vehicle or DMC (12.5–50 μM) with OSCC cells, the medium was changed to remove DMC, and SCC-9 and HSC-3 cells were respectively maintained in fresh medium for 18 and 7 days to determine the long-term death-inducing effects of DMC. Representative photomicrographs were shown in the left panel. Data was given semi-logarithmically as a survival fraction/DMC dose plot. (**D**) After 24 h treatment of vehicle or DMC (12.5–50 μM) with SCC-9 and HSC-3 cells, the cell-cycle phase distribution and cell death in the sub-G_1_ phase were analyzed by FACS after propidium iodide (PI) staining. (**E**) Diagrams summarize cell-cycle results.

Compared to CUR, the structure of DMC lacks one methoxy group directly linked to the benzene ring, as shown in Figure 1A. To investigate the pharmacological potential of DMC against OSCC, we examined short-term (24 h) and long-term treatment (8–19 days) effects of DMC on the cell growth of primary SCC-9 and metastatic HSC-3 OSCC cells, respectively using thiazolyl blue tetrazolium bromide (MTT) and colony formation assays. As shown in Figure 1B, after 24 h, DMC treatment concentration dependently inhibited the cell proliferation of both OSCC cells, and the 50% growth inhibitory concentration (IC50) was around 50 μM. We further observed that the antiproliferative ability of DMC is stronger on OSCC cells than on the normal gingival epithelial cells. In addition, the long-term growth of HSC-3 and SCC-9 cells was also significantly reduced following treatment with 12.5–50 μM of DMC, and the IC50 values were lower than 12.5 μM (Figure 1C). Based on these results, DMC can likely be useful as a therapeutic agent in managing OSCC. To further analyze the mechanism involved in DMC-induced cell growth inhibition, we next performed flow cytometry to evaluate the effect of DMC on the cell-cycle phase distribution in OSCC cells. After 24 h of DMC (12.5–50 μM) treatment in HSC-3 and SCC-9 cells, the cell cycle distribution in the G0/G1 phase had markedly attenuated, whereas the distribution of cells in the G2/M phase had markedly increased in DMC-treated cells compared to vehicle-treated cells (Figure 1D,E), suggesting that cell cycle arrest in the G2/M phase may contribute to the suppressive effects of DMC on cell viability.

### 2.2. DMC Treatment Results in the Apoptosis of OSCC Cells

**Figure 2:**
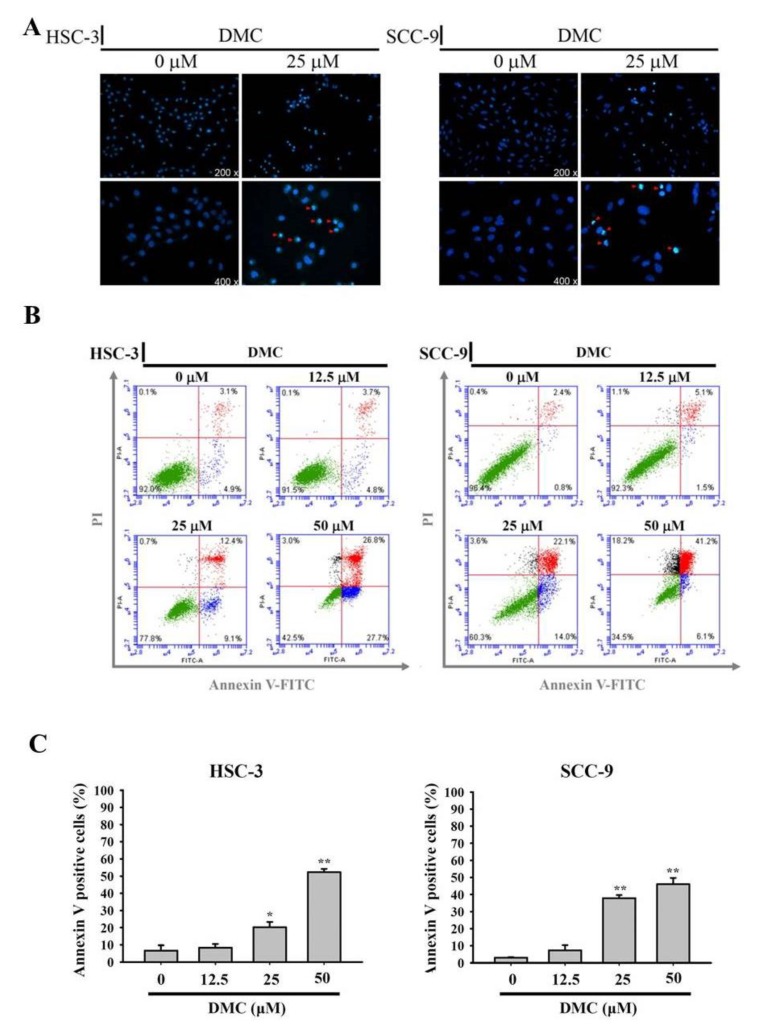
Demethoxycurcumin (DMC) induces apoptotic cell death in oral squamous cell carcinoma (OSCC) cells. (**A**) After 24 h DMC (25 μM) treatment of SCC-9 and HSC-3 cells, the morphological characteristics of apoptosis were analyzed by fluorescence microscopy after Hoechst 33342 staining. The red arrows indicated the nuclear fragmentation and condensation which served as apoptosis indicators. (**B**,**C**) Quantitative analysis of cell apoptosis by Annexin-V and propidium iodide (PI) double-staining flow cytometry in SCC-9 and HSC-3 cells treated with DMC (12.5–50 μM) or the vehicle for 24 h. One representative example of both cells is displayed in B. Values represent the mean ± SD of three independent experiments (**C**). * *p* < 0.05, ** *p* < 0.01, compared to the vehicle group.

In addition to cell-cycle arrest, an increased sub-G1 apoptotic fraction was also observed in 25 and 50 μM DMC-treated HSC-3 and SCC-9 cells (Figure 1D,E). To confirm apoptosis by cell morphological observations, HSC-3 and SCC-9 cells were treated with 25 μM DMC for 24 h, stained with Hoechst 33342, and observed by fluorescence microscopy. Morphological characteristics of apoptosis, such as nuclei with intensely bright staining and fragmented nuclei, were observed in DMC-treated cells (Figure 2A, arrows). Apoptosis induced by DMC was further checked by Annexin V-FITC/PI (propidium iodide) double-staining analysis. Figure 2B,C showed that early (PI-negative/Annexin-V-positive) and late apoptotic cells (PI-positive/Annexin-V-positive) all dramatically increased in concentration-dependent manners after treating HSC-3 and SCC-9 cells with DMC (12.5–50 μM). Percentages of total apoptotic cells treated with DMC ranged 8.9–52.2% in HSC-3 cells and 5.1–46% in SCC-9 cells (Figure 2C). These results are all hallmarks of apoptosis and demonstrated the ability of DMC to induce the apoptotic cell death of OSCC cells.

### 2.3. Targeting of cIAP1 and XIAP by DMC Triggers Caspase-Mediated Apoptotic Cell Death in OSCC

**Figure 3:**
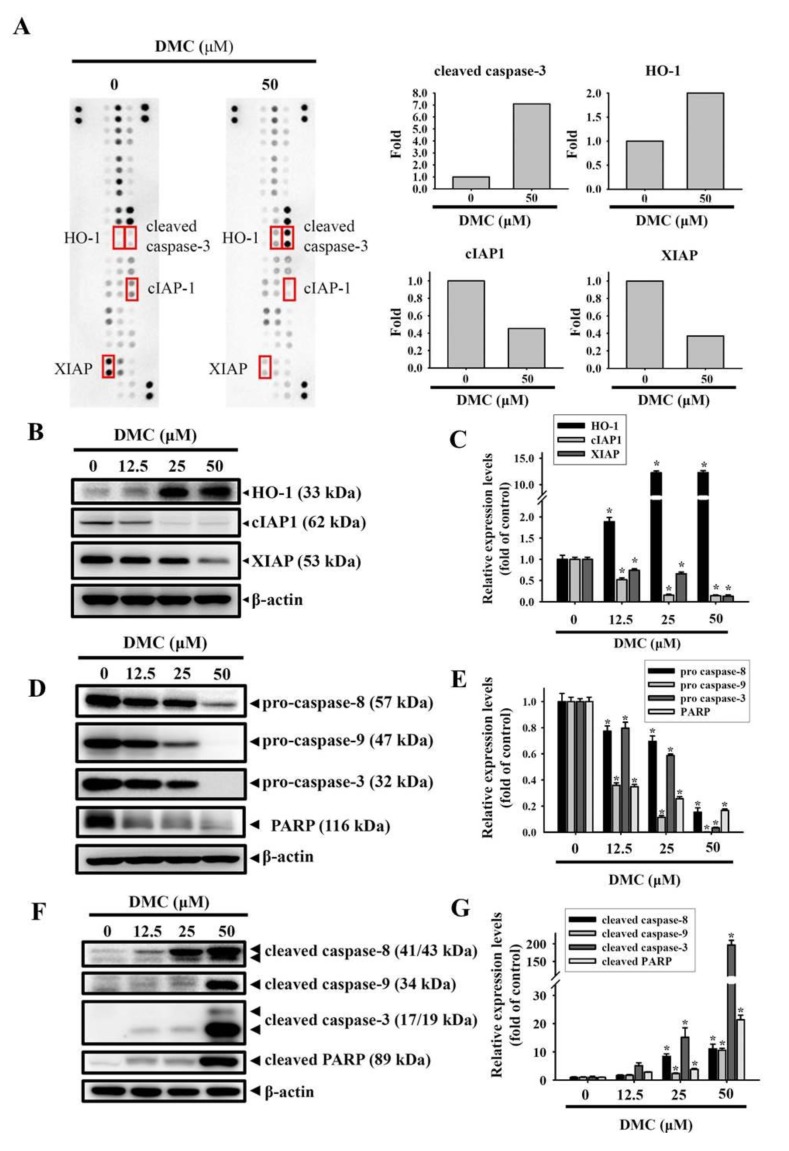
High-throughput screening of apoptosis-related proteins modulated by demethoxycurcumin (DMC) in oral squamous cell carcinoma (OSCC) cells. (**A**, left panel) Representative images of the apoptotic protein array (R&D System) are shown for vehicle- and DMC-treated HSC-3 cells. (**A**, right panel) Proteins involved in apoptosis and regulatory pathways were quantitated using a densitometer and are represented as multiples of change compared to the controls. (**B**–**G**) HSC-3 cells were treated with indicated concentrations of DMC for 24 h, and a Western blot analysis was used to detect the expression levels of heme oxygenase (HO)-1, cellular inhibitor of apoptosis 1 (cIAP1), X-chromosome-linked IAP (XIAP), pro- and cleaved caspases-3, -8, and -9, and poly(ADP-ribose) polymerase (PARP) (**B**,**D**,**F**). The β-actin protein levels were used to adjust the quantitative results of these protein levels and expressed as multiples of induction beyond each respective control. Values are presented as mean ± SD from three independent experiments. * *p* < 0.05, compared to the vehicle group (**C**,**E**,**G**).

To gain insights into the mechanism of apoptosis induced by DMC in HSC-3 OSCC cells, we evaluated levels of proteins involved in regulating apoptosis using a human apoptosis array (ARY009, R&D Systems), which contained 35 different apoptosis-related proteins. Several apoptosis-related proteins were respectively upregulated and downregulated in DMC-treated HSC-3 cells compared to vehicle-treated cells (Figure 3A). We next validated the results by a Western blot analysis and found that DMC treatment respectively induced an increase in the HO-1 protein and decreases in cIAP1/XIAP proteins in concentration-dependent manners (Figure 3B,C). XIAP and cIAP1 were reported to inhibit intrinsic and extrinsic apoptosis through directly and indirectly inducing the inactivation of caspases-3, -9, and -8 [27]. Herein, the exposure of HSC-3 cells to DMC (12.5–50 μM for 24 h) concentration-dependently induced the degradation of procaspases-8, -9, and -3, which respectively generated active forms of caspases-8, -9, and -3 (Figure 3D–G). The cleavage of poly(ADP-ribose) polymerase (PARP) by caspase-3 was also concentration-dependently induced by DMC treatment (Figure 3F,G). In addition to HSC-3 cells, the downregulation of cIAP and XIAP, upregulation of HO-1, and activation of caspases-8/-9/-3 were also observed in DMC-treated SCC-9 cells (Figure S1). These findings suggest that the inhibition of cIAP1 and XIAP was responsible for the DMC-induced caspase-mediated apoptotic cell death of OSCC cells.

### 2.4. HO-1 Is a Critical Upstream Regulator Involved in DMC-Induced Caspase-Mediated Apoptotic Cell Death in OSCC Cells

**Figure 4:**
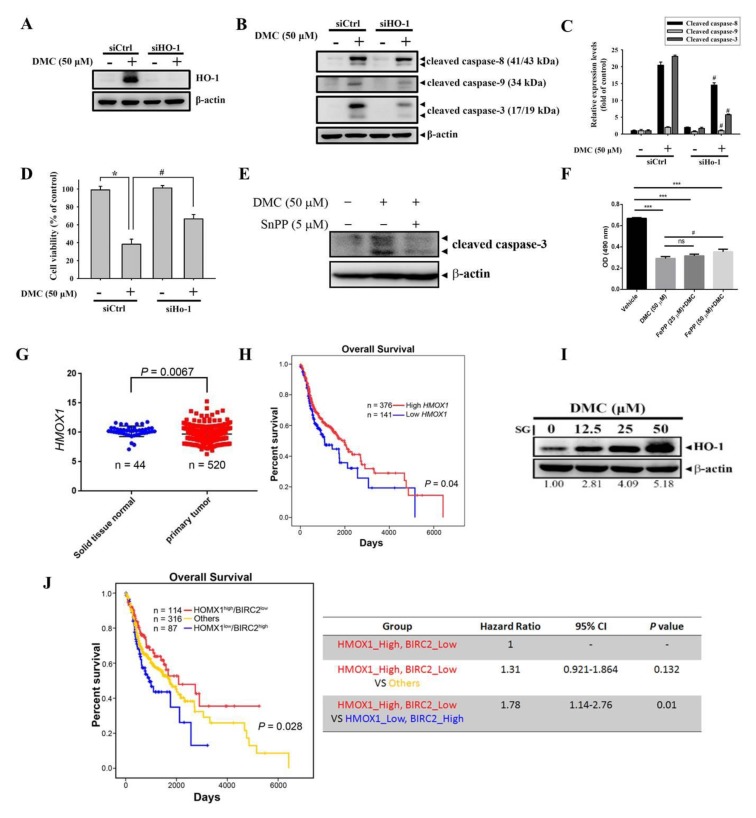
Heme oxygenase (HO)-1 is an upstream regulator involved in demethoxycurcumin (DMC)-induced caspase activation and the subsequent induction of apoptosis in oral squamous cell carcinoma (OSCC) cells. (**A**–**D**) HSC-3 cells were transiently transfected with HO-1-specific siRNA or control siRNA (siCtrl) and subjected to Western blot and MTT assays. The knockdown efficiency of HO-1 siRNA is shown in A. HO-1-specific siRNA reversed the DMC-induced increases in cleaved caspases-3, -8, and -9 (**B**,**C**) and the decrease of cell proliferation (**D**) of HSC-3 cells. Data are presented as the mean ± SD of three independent experiments. * *p* < 0.05, compared to the vehicle group; ^#^ *p* < 0.05, compared to the siCtrl-transfected group. (**E**) Enzyme activity of HO-1 is essential for the pro-apoptotic effect of DMC in OSCC cells. SCC-9 cells were treated with DMC in the presence or absence of the HO-1 enzymatic inhibitor, SnPP (5 μM), for 24 h, and the expression of cleaved caspase-3 was analyzed by a Western blotting analysis. (**F**) The effect of combined treatment with DMC and iron protoporphyrin IX (FePP)/heme on cell viability of OSCC cells. SCC-9 cells were treated with DMC (50 μM) simultaneously with or without FePP (25 or 50 μM) for 24 h and then subjected to MTS assay to determine the cell viability. Columns, mean (*n* = 3); bars, SD. *** *p* < 0.001 compared with the vehicle group. ^#^ *p* < 0.05 compared with the DMC-treated only group. ns: not significant. (**G**) Expressions of mRNA levels of *HMOX1* (FPKM) in normal tissues (*n* = 44) and primary head–neck tumors (*n* = 520). (**H**) Correlation of *HMOX1* expression and overall survival (OS) in head–neck squamous cell carcinoma using a Kaplan–Meier analysis. (**I**) SG cells were treated with indicated concentrations of DMC for 24 h, and a Western blot analysis was used to detect expression levels of HO-1. (**J**) All patients were separated into a negative correlation of *HMOX1* and *BIRC2* expression, low *HMOX1* and high *BIRC2* (H0B1), and high *HMOX1* and low *BIRC2* (H1B0), and others. Data showed that patients in the H1B0 group had the most favorable prognosis (overall *p*-value of 0.028). In the negative-correlated groups, patients in the H0B1 group had a worse prognosis than those in the H1B0 group (*p* = 0.01). The head–neck cancer dataset was retrieved from The Cancer Genome Atlas (TCGA).

To further determine the role of upregulated HO-1 induced by DMC in DMC-mediated growth inhibition and apoptosis in OSCC cells, we knocked down HO-1 with HO-1-specific siRNA. We observed that the transfection of HO-1-specific siRNA significantly reversed the DMC-induced increase of HO-1 protein (Figure 4A), with concomitant decreases in the activation of caspases-8/-9/-3 in DMC-treated HSC-3 cells compared to control siRNA-transfected cells (Figure 4B,C). Moreover, the silencing of HO-1 significantly rescued DMC-mediated growth inhibition (Figure 4D). To further investigate whether the enzyme activity of HO-1 was involved in the pro-apoptotic effect of DMC, a HO-1 enzymatic inhibitor, tin protoporphyrin (SnPP) was used. We found the induction of caspase-3 activation by DMC in SCC-9 cells was reversed by the SnPP pretreatment (Figure 4E). Moreover, we observed that in the presence of different concentrations of iron protoporphyrin IX (FePP)/heme can result in non-significant or partial levels of protection against the antiproliferative effect of DMC in SCC-9 cells (Figure 4F). In the clinic, we analyzed HO-1 gene (*HMOX1*) expression data obtained from The Cancer Genome Atlas (TCGA) and found that significantly lower *HMOX1* transcripts were observed in head and neck tumors, compared to normal tissues (Figure 4G). The Kaplan–Meier (KM) plot revealed a longer overall survival of head and neck cancer patients with high HO-1 (*HMOX1*) expression than patients with low HO-1 expression (*p* = 0.04; Figure 4H). These data suggest that upregulating HO-1 is crucial for DMC-induced caspase-mediated apoptotic cell death, and that high HO-1 levels predict a favorable prognosis in patients with head and neck cancer. In comparison with OSCC cells (Figure 3C), the inducible level of HO-1 by DMC is lower in normal gingival epithelial cells, SG (Figure 4I), suggesting that this might be the reason DMC exerts less toxicity on normal oral epithelial cells. 

Furthermore, from the same TCGA database described above, patients with head and neck tumors with *HMOX1*^high^/*BIRC2*^low^ had the longest survival times compared to those with *HMOX1*^low^/*BIRC2*^high^, *HMOX1*^high^/*BIRC2*^high^, or *HMOX1*^low^/*BIRC2*^low^ (Figure 4J). Clinical data indicated that the upregulation of HO-1 and downregulation of cIAP-1 are critical events in retarding the progression of head and neck cancers.

### 2.5. Activation of the p38 MAPK-HO-1 Signaling Cascade by DMC Triggers Caspase-Mediated Apoptotic Cell Death in OSCC Cells

**Figure 5:**
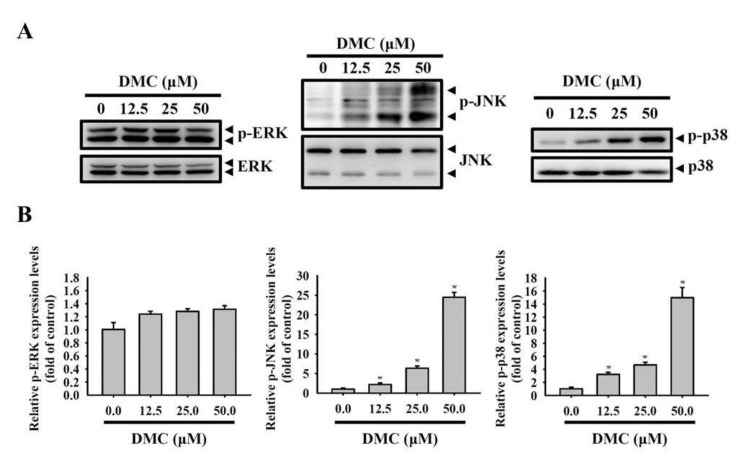

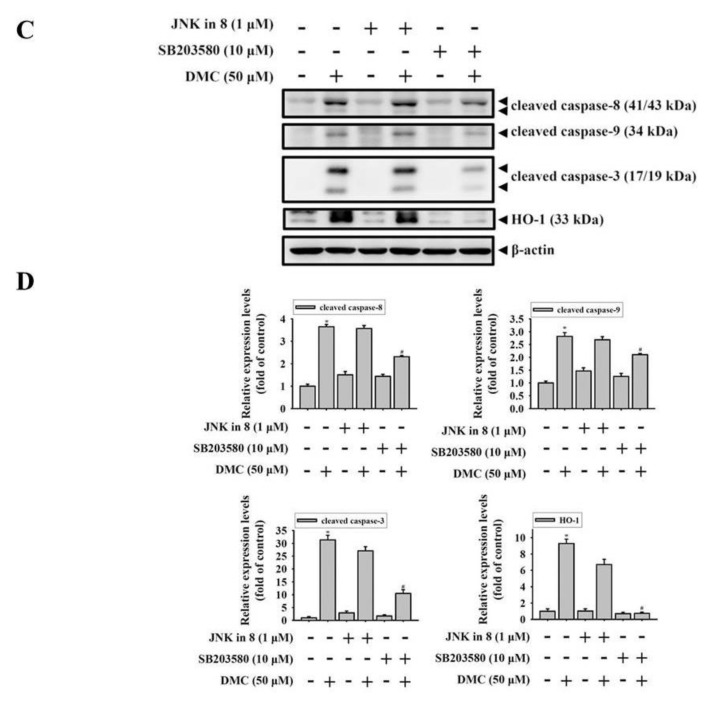
The p38 mitogen-activated protein kinase (MAPK) pathway is involved in the demethoxycurcumin (DMC)-mediated induction of heme oxygenase (HO)-1 expression and cell apoptosis. (**A**,**B**) HSC-3 cells were exposed to the vehicle or DMC (12.5–50 μM) for 24 h; then, the phosphorylation status of extracellular signal-regulated kinase (ERK)1/2, c-Jun N-terminal kinase (JNK)1/2, and p38 were analyzed by Western blot analysis (**A**). Quantitative results of phospho-MAPKs, which were adjusted to total MAPKs and are expressed as multiples of induction beyond each respective control (**B**). Data are presented as the mean ± SD from three independent experiments. * *p* < 0.05, compared to the vehicle group. (**C**,**D**) HSC-3 cells were pretreated with SB203580 (10 μM) or c-Jun N-terminal kinase (JNK)-in-8 (1 μM) for 1 h followed by another 24-h vehicle or DMC (50 μM) treatment. Levels of cleaved caspase-3, -8, and -9, and HO-1 were analyzed by a Western blot analysis (**C**). Quantitation of Western blots normalized to β-actin was carried out using Image-pro plus processing software (**D**). Data are presented as the mean ± SD of three independent experiments. * *p* < 0.05, compared to the vehicle group; ^#^ *p* < 0.05, compared to the DMC-treated group.

Previous studies showed that the MAPK signaling pathway plays an important role in the CUR-mediated apoptosis of diverse cancer types [28]. HO-1 is one of the proteins regulated by mitogen-activated protein kinase (MAPK) signaling systems [29,30], but the relationship between MAPK signaling and HO-1 after DMC exposure has not yet been elucidated. Therefore, we examined whether DMC can induce the activation of three major MAPKs including extracellular signal-regulated kinase (ERK), c-Jun N-terminal kinase (JNK), and p38. As shown in Figure 5A,B, DMC significantly activated JNK1/2 and p38 MAPK, but not ERK1/2, in concentration-dependent manners. To further determine the role of JNK1/2 and p38 MAPK activation in DMC-induced HO-1 upregulation and cell apoptosis, 1 h of pretreatment of 10 μM SB203580 (a p38 inhibitor) or 1 μM JNK-in-8 (a JNK inhibitor) with HSC-3 cells was followed by 50 μM DMC treatment for another 24 h, and then cells were subjected to Western blotting analysis (Figure 5C). Our results revealed that only the inhibition of p38 MAPK in HSC-3 cells considerably reversed DMC-induced HO-1 expression and caspase-8/-9/-3 activation (Figure 5D). A similar phenomenon was also observed in SCC-9 cells (Figure S2). Overall, these results suggest that DMC induces caspase-mediated cell apoptosis through activating the p38 MAPK-HO-1 signaling cascade in OSCC cells.

## 3. Discussion

Accumulating evidence has shown that DMC is a more effective and stable curcuminoid than CUR or BDMC in cancer treatment of prostate, lung, and brain tumors [24,31,32] and other diseases [33]. In the present study, we observed that DMC exhibited strong oncostatic effects on OSCC cells respectively derived from primary and metastatic sites, including G_2_/M cell-cycle arrest and apoptotic cell death. Upregulation of the p38 MAPK-HO-1 axis and downregulation of cIAP1/XIAP were critical for DMC-induced apoptotic cell death in OSCC cells.

G2/M cell-cycle arrest is one of the most prominent checkpoints of many anticancer agents, which can induce proliferation inhibition and apoptosis by suppressing the segregation of damaged chromosomes during mitosis [34]. The present study first revealed that the exposure of OSCC cells to DMC resulted in an increased percentage of cells in the G_2_/M phase together with a decrease in the G_0_/G_1_ phase. Meanwhile, an increase in the sub-G_1_ peak, which is a characteristic feature of cell apoptosis, was also induced by DMC, suggesting that G_2_/M arrest is an underlying mechanism inhibiting the growth of DMC-treated OSCC cells, which might further turn on an apoptotic program. These results are supported by other published studies which indicate that DMC markedly induced G_2_/M arrest in brain and prostate cancers [24,35]. Evidence in the literature indicates that DMC induced BCL-2-mediated G_2_/M arrest most effectively among CUR, DMC, and BDMC in brain tumors [24]. In addition, other positive regulators that participate in the G_2_/M transition, such as CDC25C phosphatase and cyclin B1, were downregulated by DMC through inducing reactive oxygen species (ROS) production in brain tumor cells [36]. Although the DMC-induced increase in ROS in OSCC cells was recently reported [37], the effect of DMC on these regulators of the G_2_/M transition needs to be further investigated in the future.

The IAP family, particularly cIAP1, cIAP2, XIAP, and survivin, are proteins that have substantial roles in modulating the inactivation of apoptosis and overexpression in OSCC [38]. Among these IAP proteins, the overexpression of XIAP or cIAP1 was reported to be correlated with a poor prognosis and chemoresistance in head and neck cancer [6,7]. Hence, a cIAP-targeting or cIAP/XIAP dual-targeting therapeutic approach was recently demonstrated as a potential strategy for treating head and neck cancers. Moreover, some IAP inhibitors are currently in clinical trials as monotherapy or combination therapy with chemotherapeutic drugs or radiotherapy in different solid tumors, including head and neck [27]. The apoptosis array in our present study showed that DMC can suppress the expressions of cIAP1 and XIAP in OSCC cells, suggesting that DMC might be a potential dual antagonist of cIAP/XIAP. Actually, another dual antagonist of cIAP/XIAP, ASTX660, was reported to sensitize head and neck cancer to tumor necrosis factor (TNF) family death ligands (such as TNF and TRAIL) and radiation [9]. Moreover, the nuclear factor-kappa B (NF-κB) transcription factor was found to be constitutively activated in OSCC [39] and shown to regulate expressions of cIAP1 and XIAP [40]. Constitutive NF-κB activation has been attributed to a lack of response of SCC-9 OSCC cells to TNF-α [41]. Furthermore, DMC was recently shown to inhibit NF-κB activity in OSCC cells [37]. Taken together, DMC might enhance the therapeutic effect of chemotherapeutic agents in OSCC treatment via targeting NF-κB-mediated IAP expression, and this hypothesis is worthy of further investigation in the future.

In addition to cIAP1/XIAP targeting, DMC was found to significantly induce the upregulation of HO-1 from the apoptosis array in OSCC cells. Actually, HO-1 was previously reported to be induced by CUR in diverse cell types and play various roles in different cell types. For example, HO-1 was induced by CUR to exert its antiproliferative effect in vascular smooth muscle cells [42]. In human monocytes, CUR-induced HO-1 expression showed its anti-inflammatory effects [43]. In cancer treatment with CUR, HO-1 induction also plays a conflicting role in different cancer types. In breast cancer, the induction of HO-1 and its catalyzed byproduct, CO, by CUR can attenuate heat shock protein (HSP) 90 activity and its client proteins Akt, CDK4, and cyclinD1 to further suppress the invasion and proliferation of cells [44]. In colorectal cancer (CRC), a pro-apoptotic effect of HO-1 was observed in CRC cells via the induction of CO and endoplasmic reticular (ER) stress [45]. In contrast, CUR-induced HO-1 played a negative role for its anticancer effect in bladder cancers [46]. Although CO from the heme degradation reaction catalyzed by HO-1 is previously known to have anti-apoptotic functions [47], the recent studies mentioned above indicated that heme-mediated CO production also can induce a pro-apoptotic effect in cancer cells. The opposite role of CO in regulating cell apoptosis might be due to the amount of CO production. Low doses of CO were reported to prevent apoptosis in different cell models [48]. In contrast, a moderately high concentration of CO exerts pro-apoptotic effects toward several cell types, including cancer cells [49]. The production of CO catalyzed by HO-1 was reported to further induce HO-1 expression in cancer cells [44], and this positive feedback loop can promote a high amount of CO production. 

As for the role of HO-1 in OSCC, previous studies indicated that the HO-1 expression level was negatively correlated with lymph node metastasis in OSCC patients [18]. Herein, we also observed that head and neck cancer patients harboring high HO-1 (*HMOX1*) expression in tumor tissues had significantly more favorable overall survival than those with a lower level. Moreover, we observed that DMC can induce HO-1 upregulation to mediate caspase-dependent apoptosis in OSCC cells, suggesting that DMC-induced HO-1 plays a positive role in its anticancer effect in OSCC. Our present study has shown that the induction of caspase-3 activation by DMC was reversed by the HO-1 enzymatic inhibitor, SnPP, pretreatment, suggesting that the HO-1 enzymatic activity is essential for the pro-apoptotic effect of DMC. Moreover, different concentrations of FePP/heme were shown to induce non-significant or partial levels of protection against the anti-proliferative effect of DMC in OSCC cells. Actually, a previous study has indicated that the free iron-induced upregulation of ferritin plays a critical role in protecting the CUR-induced apoptosis of keratinocytes [50]. In contrast, CUR was also reported to act as an iron chelator to interfere with ferritin expression and induce the apoptosis of prostate cancer cells [51]. Furthermore, the in vivo anticancer activities of DMC have been documented in a xenograft brain tumor-bearing mice model [26], suggesting the expression level of heme in brain tumors might not effectively abrogate the anticancer effect of DMC. These results suggest that the anticancer efficacy of DMC in various cancers in vivo might be dependent on the different proportions of heme-derived free iron and DMC in tumor tissues. In addition to free iron, the roles of HO-1-catalyzed CO and other catalyzed byproducts from heme in the anticancer activity of DMC on OSCC should be further investigated in the future. We next investigated the DMC-mediated signal transduction in regulating HO-1 expression. On the basis of previous reports that MAPKs are involved in CUR-mediated HO-1 expression [52], a panel of kinase inhibitors was used to dissect the contribution of MAPKs to the DMC-mediated upregulation of HO-1. We observed that p38 MAPK activity was essential for HO-1 expression induced by DMC.

Available data showed that more than 80% oral cancer patients and oral cancer cell lines exhibit an overexpression of EGFR. Actually, the OSCC cell lines we used here also expressed EGFR (Figure S3). Various strategies have been developed to disrupt EGFR function for OSCC treatment such as anti-EGFR antibody (cetuximab) and EGFR tyrosine kinase inhibitors (TKIs) (gefitinib) [53]. Recently, a combination of cetuximab with chemotherapy such as cisplatin, 5-fluorouracil, docetaxel, or paclitaxel has become the new standard advanced treatment for OSCC [54,55]. Actually, a previous report has screened almost 600 herbal and natural compounds and found that CUR could promote EGFR degradation to potentiate the inhibitory effect of gefitinib on gefitinib-resistant lung cancer cells in vitro and in vivo [56]. Moreover, synergistic inhibitory effects of cetuximab and CUR on cisplatin-resistant oral cancer cells have also been documented recently [57]. Furthermore, after screening 36 CUR analogues, DMC was demonstrated to show the best inhibitory effects on both wild-type and mutant EGFR [58]. In fact, we also observed that DMC can inhibit EGFR expression in OSCC cells (Figure S4A) and further enhance the inhibitory effect of gefitinib on cell proliferation (Figure S4B), suggesting that the enhancement of DMC on gefitinib-mediated growth inhibition of OSCC cells might be through inducing EGFR degradation. In addition to targeting EGFR, another CUR analogue, BDMC, was reported to promote the suppressive effect of PDL-1 antibody on bladder cancer progression via stimulating cytotoxic T-cell activity and suppressing myeloid-derived suppressor cells (MDSCs) in an immunocompetent mice model [59], suggesting that DMC might be also an immunomodulatory compound in the tumor microenvironment. According to these observations, we suggested that the combination of DMC with cetuximab, gefitinib, or a PDL-1 antibody might be a good treatment strategy for advanced OSCC. 

## 4. Materials and Methods

### 4.1. Cell Lines and Reagents

The human OSCC lines SCC-9 and HSC-3 were respectively derived from primary and metastatic sites of tongue squamous cell carcinoma and were obtained from the American Type Culture Collection (Manassas, VA, USA). The Smulow–Glickman (SG) human gingival epithelial cell line was original from F.H. Kasten, East Tennessee State University, Quillen College of Medicine, Johnson City, TN. Culture conditions of both OSCC cell lines were maintained in Dulbecco’s Modified Eagle Medium/Ham’s F12 Nutrient Mixture (DMEM/F12; Life Technologies, Grand Island, NY, USA) supplemented with 10% fetal bovine serum (FBS) (Gibco, Grand Island, NY, USA) and other essential supplements as previously described [60]. The SG cells were cultured in DMEM medium supplemented with 10% FBS. 

DMC of 98% purity, dimethyl sulfoxide (DMSO), SnPP, Hoechst 33342, and thiazolyl blue tetrazolium bromide (MTT) were purchased from Sigma Chemical (St. Louis, MO, USA). Propidium iodide (PI) was obtained from Invitrogen (Carlsbad, CA, USA). JNK-in-8 (a JNK1/2 inhibitor) and SB203580 (a p38 inhibitor) were purchased from Calbiochem (San Diego, CA, USA). The primary antibodies against cleaved Caspase-8 (#9496), cleaved Caspase-9 (#9505), cleaved Caspase-3 (#9664), Caspase-8 (#9746), Caspase-9 (#9502), PARP (#9542), Phospho-Erk1/2 (#4370), Erk1/2 (#9102), Phospho-JNK (#4668), JNK2 (#9258), Phospho-EGFR (#2220), EGFR (#2239), c-IAP1 (#7065), and XIAP (#2045) were obtained from Cell Signaling Technology (Danvers, MA, USA). Anti-Caspase-3 (610323), anti-phospho-p38 (612281), and anti-p38 (612168) were purchased from BD biosciences (San Jose, CA, USA). Anti-β-actin (ab8226) and anti-HO-1 (ab68477) were purchased from Abcam (Cambridge, UK). Anti-mouse IgG (5450-0011) and anti-rabbit (5450-0010) secondary antibodies were purchased from Seracare life sciences (Milford, MA, USA).

### 4.2. Cell Viability Assay

The cytotoxic effect of DMC on cell viability was measured by an MTT assay-based colorimetric assay. Briefly, OSCC and SG cells were plated in 24-well plates for 24 h of incubation and treated with indicated concentrations of DMC (0, 12.5, 25, and 50 μM) for another 24 h. After washing DMC out of cells, MTT (0.5 mg/mL) was added to the culture medium for 4 h at 37 °C. Finally, the amount of the MTT formazan product was dissolved in isopropanol, and absorbance values were measured by a microplate reader (MQX200; Bio-Tek Instruments, Winooski, VT, USA) at 563 nm.

### 4.3. Plate Colony-Formation Assay

OSCC cells (10^3^) were plated in six-well plates and incubated for 24 h. Subsequently, cells were treated with DMC at the indicated concentrations (0, 12.5, 25, and 50 μM) and incubated for another 24 h at 37 °C. Thereafter, the medium was changed to remove DMC, and 7–18 days later, cells were stained with crystal violet. Colonies were manually counted using ImageJ free software (National Institutes of Health, Bethesda, MD, USA).

### 4.4. Cell-Cycle Distribution Assay

DMC-induced changes in the cell-cycle distribution were analyzed by flow cytometry (Beckman Coulter, Los Angeles, CA, USA). After treatment, OSCC cells were washed with phosphate-buffered saline (PBS), fixed with ice-cold 70% ethanol at ‒20 °C for 12 h, and stained with PI buffer including RNase A (100 μg/mL). DNA contents of stained cells were determined by a FACScan laser flow cytometric analysis system. The proportion of nuclei in each phase of the cell cycle was analyzed, and apoptotic cells with a hypodiploid DNA peak were detected in the sub-G_1_ region.

### 4.5. Apoptosis Assays

Apoptotic cell death induced by DMC was determined following the manufacturer’s guidelines of the FITC-labeled Annexin-V/PI Apoptosis Detection kit (BD Biosciences, San Jose, CA, USA). After treatment, OSCC cells were washed twice with PBS, resuspended in binding buffer (10 mM HEPES, 140 mM NaCl, and 2.5 mM CaCl_2_ at pH 7.4), and stained with 5 μL FITC-conjugated Annexin-V and 5 μL of PI for 20 min in the dark. Data acquisition and analysis were performed in a Becton–Dickinson FACSCalibur flow cytometer using CellQuest software (BD Biosciences).

### 4.6. Nuclear Morphological Analysis by Hoechst 33342

DMC-induced morphological changes in the nuclear chromatin of cells were visualized following DNA staining using Hoechst 33342. After treatment, OSCC cells were fixed with 4% formaldehyde solution for 15 min, incubated for 10 min in a Hoechst 33,342 solution, and examined using a Zeiss Axiophot fluorescence microscope (Carl Zeiss Microimaging, Gottingen, Germany). Morphological features of apoptotic cells comprised chromatin condensation and nuclear fragmentation.

### 4.7. Human Apoptosis Proteome Profiler Array

To clarify the pathways through which DMC induced apoptosis, we determined apoptosis-related proteins using the Proteome Profiler Human Array (R&D Systems, Minneapolis, MN, USA). After treatment, cell lysate samples (200 μg) were extracted and applied per array set comprised of two nitrocellulose membranes with spotted capture antibodies. Next, a biotinylated antibody cocktail and streptavidin–horseradish peroxidase were used to detect the bound material using chemiluminescence detection. The pixel density of spots was quantified using Image-Pro Plus software. Spot densities were normalized against respective reference array spots and then against controls.

### 4.8. Protein Lysate Preparation and Western Blot Analysis

The preparation of protein lysates and performance of the Western blot analysis followed previously described procedures [61]. Briefly, OSCC cells were lysed and extracted by radioimmunoprecipitation assay (RIPA) buffer (Sigma-Aldrich, St. Louis, MO, USA). A Western blot analysis was performed with indicated primary antibodies and horseradish peroxidase-conjugated secondary antibodies. After washing, blots were incubated with the ECL reagent (Millipore, Billerica, MA, USA), and the protein expression was detected by chemiluminescence.

### 4.9. Small Interfering (SI)RNA Transfection

*HMOX1* gene silencing was performed using siRNAs targeting *HMOX1* (#4390824, s6674 Ambion) and a negative control (#4390844, Ambion). Lipofectamine RNAiMAX Transfection reagent (Thermo Fisher Scientific, Waltham, MA, USA) was used to transfect each siRNA (150 pmol) into OSCC cells following the manufacturer’s guidelines.

### 4.10. Bioinformatics Analysis

A clinical analysis of the molecular expression by RNAseq in a head and neck cancer patient cohort was obtained from The Cancer Genome Atlas (TCGA) UCSC Xena website (https://xenabrowser.net/). The prognostic significance of *HMOX1* and *BIRC2* levels in 517 head and neck cancer patients was examined using a Kaplan–Meier analysis with the best cutoff threshold.

### 4.11. Statistical Analysis

Values are shown as the mean ± standard deviation (SD) from three independent experiments. Statistical analyses were performed using SigmaPlot, vers. 10.0 (Systat Software, SigmaPlot for Windows). A non-parametric test was used for comparisons between two groups due to the small sample size. Differences were considered significant at *p* values of < 0.05.

## 5. Conclusions

**Figure 6:**
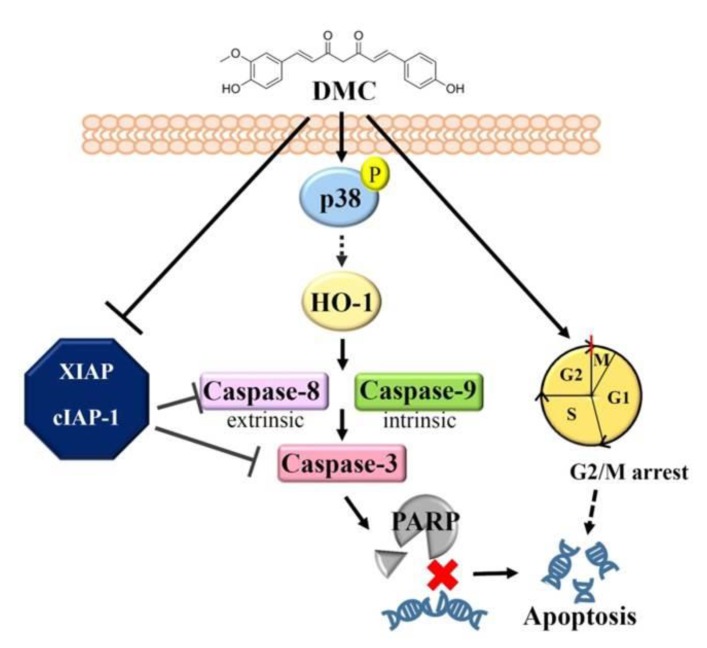
A working model shows the molecular mechanism underlying the ability of demethoxycurcumin (DMC) to suppress the growth of oral squamous cell carcinoma (OSCC) cells. The antiproliferative activity of DMC against OSCC cells derived from primary and metastatic sites was attributed to inhibition of cellular inhibitor of apoptosis 1 (cIAP1)/X-chromosome-linked IAP (XIAP) expression and activation of the p38 mitogen-activated protein kinase (MAPK)-heme oxygenase (HO)-1 axis, with the ultimate induction of apoptotic cell death. Moreover, the induction of G2/M arrest might be another cause for the DMC-induced apoptotic cell death in OSCC cells.

In summary, our data demonstrate for the first time that DMC is effective at suppressing the expression of cIAP1/XIAP and activating the p38 MAPK-HO-1 axis, resulting in the intrinsic and extrinsic apoptotic cell death of OSCC cells. Moreover, the cell apoptotic effect was also possibly contributed by G_2_/M cycle arrest induced by DMC, and the mechanism is schematically illustrated in Figure 6. In the clinic, patients with head and neck tumors and *HMOX1*^high^/*BIRC2*^low^ had the most favorable prognosis. Therefore, our present findings strongly support the development of clinical trials to determine whether DMC or DMC combined with other chemotherapeutic drug regimens would be useful in managing human OSCC.

## Supplementary Materials

The following are available online at https://www.mdpi.com/2072-6694/12/3/703/s1, Figure S1: Effect of demethoxycurcumin (DMC) on apoptosis-related proteins in oral squamous cell carcinoma (OSCC) cells, Figure S2: Activation of p38 mitogen-activated protein kinase (MAPK) is involved in demethoxycurcumin (DMC)-induced heme oxygenase (HO)-1 expression and cell apoptosis in SCC9 cells, Figure S3: Levels of endogenous epidermal growth factor receptor (EGFR) were analyzed by Western blot analysis in HSC-3 and SCC-9 oral squamous cell carcinoma (OSCC) cells, Figure S4: Demethoxycurcumin (DMC) potentiates the growth inhibitory effect of gefitinib on oral squamous cell carcinoma (OSCC) cells.

## References

[1] Torre L.A., Bray F., Siegel R.L., Ferlay J., Lortet-Tieulent J., Jemal A. (2015). Global cancer statistics, 2012. CA Cancer J. Clin..

[2] Le Campion A., Ribeiro C.M.B., Luiz R.R., da Silva Junior F.F., Barros H.C.S., Dos Santos K.C.B., Ferreira S.J., Goncalves L.S., Ferreira S.M.S. (2017). Low Survival Rates of Oral and Oropharyngeal Squamous Cell Carcinoma. Int. J. Dent..

[3] Sasahira T., Kirita T. (2018). Hallmarks of Cancer-Related Newly Prognostic Factors of Oral Squamous Cell Carcinoma. Int. J. Mol. Sci..

[4] Nagata M., Nakayama H., Tanaka T., Yoshida R., Yoshitake Y., Fukuma D., Kawahara K., Nakagawa Y., Ota K., Hiraki A. (2011). Overexpression of cIAP2 contributes to 5-FU resistance and a poor prognosis in oral squamous cell carcinoma. Br. J. Cancer.

[5] Qi S., Mogi S., Tsuda H., Tanaka Y., Kozaki K., Imoto I., Inazawa J., Hasegawa S., Omura K. (2008). Expression of cIAP-1 correlates with nodal metastasis in squamous cell carcinoma of the tongue. Int. J. Oral Maxillofac. Surg..

[6] Tanimoto T., Tsuda H., Imazeki N., Ohno Y., Imoto I., Inazawa J., Matsubara O. (2005). Nuclear expression of cIAP-1, an apoptosis inhibiting protein, predicts lymph node metastasis and poor patient prognosis in head and neck squamous cell carcinomas. Cancer Lett..

[7] Yang X.H., Feng Z.E., Yan M., Hanada S., Zuo H., Yang C.Z., Han Z.G., Guo W., Chen W.T., Zhang P. (2012). XIAP is a predictor of cisplatin-based chemotherapy response and prognosis for patients with advanced head and neck cancer. PLoS ONE.

[8] Scheurer M.J.J., Seher A., Steinacker V., Linz C., Hartmann S., Kubler A.C., Muller-Richter U.D.A., Brands R.C. (2019). Targeting inhibitors of apoptosis in oral squamous cell carcinoma in vitro. J. Craniomaxillofac Surg..

[9] Xiao R., An Y., Ye W., Derakhshan A., Cheng H., Yang X., Allen C., Chen Z., Schmitt N.C., Van Waes C. (2019). Dual Antagonist of cIAP/XIAP ASTX660 Sensitizes HPV(−) and HPV(+) Head and Neck Cancers To TNFalpha, TRAIL, and Radiation Therapy. Clin. Cancer Res..

[10] Gozzelino R., Jeney V., Soares M.P. (2010). Mechanisms of cell protection by heme oxygenase-1. Annu. Rev. Pharmacol. Toxicol..

[11] Podkalicka P., Mucha O., Jozkowicz A., Dulak J., Loboda A. (2018). Heme oxygenase inhibition in cancers: Possible tools and targets. Contemp. Oncol. (Pozn).

[12] Berberat P.O., Dambrauskas Z., Gulbinas A., Giese T., Giese N., Kunzli B., Autschbach F., Meuer S., Buchler M.W., Friess H. (2005). Inhibition of heme oxygenase-1 increases responsiveness of pancreatic cancer cells to anticancer treatment. Clin. Cancer Res..

[13] Was H., Cichon T., Smolarczyk R., Rudnicka D., Stopa M., Chevalier C., Leger J.J., Lackowska B., Grochot A., Bojkowska K. (2006). Overexpression of heme oxygenase-1 in murine melanoma: Increased proliferation and viability of tumor cells, decreased survival of mice. Am. J. Pathol..

[14] Ciesla M., Marona P., Kozakowska M., Jez M., Seczynska M., Loboda A., Bukowska-Strakova K., Szade A., Walawender M., Kusior M. (2016). Heme Oxygenase-1 Controls an HDAC4-miR-206 Pathway of Oxidative Stress in Rhabdomyosarcoma. Cancer Res..

[15] Zou C., Zou C., Cheng W., Li Q., Han Z., Wang X., Jin J., Zou J., Liu Z., Zhou Z. (2016). Heme oxygenase-1 retards hepatocellular carcinoma progression through the microRNA pathway. Oncol. Rep..

[16] Hill M., Pereira V., Chauveau C., Zagani R., Remy S., Tesson L., Mazal D., Ubillos L., Brion R., Asghar K. (2005). Heme oxygenase-1 inhibits rat and human breast cancer cell proliferation: Mutual cross inhibition with indoleamine 2,3-dioxygenase. FASEB J..

[17] Skrzypek K., Tertil M., Golda S., Ciesla M., Weglarczyk K., Collet G., Guichard A., Kozakowska M., Boczkowski J., Was H. (2013). Interplay between heme oxygenase-1 and miR-378 affects non-small cell lung carcinoma growth, vascularization, and metastasis. Antioxid. Redox Signal..

[18] Yanagawa T., Omura K., Harada H., Nakaso K., Iwasa S., Koyama Y., Onizawa K., Yusa H., Yoshida H. (2004). Heme oxygenase-1 expression predicts cervical lymph node metastasis of tongue squamous cell carcinomas. Oral Oncol..

[19] Chen H.W., Huang H.C. (1998). Effect of curcumin on cell cycle progression and apoptosis in vascular smooth muscle cells. Br. J. Pharmacol..

[20] Diaz Osterman C.J., Gonda A., Stiff T., Sigaran U., Valenzuela M.M., Ferguson Bennit H.R., Moyron R.B., Khan S., Wall N.R. (2016). Curcumin Induces Pancreatic Adenocarcinoma Cell Death Via Reduction of the Inhibitors of Apoptosis. Pancreas.

[21] Sahin K., Orhan C., Tuzcu M., Sahin N., Tastan H., Ozercan I.H., Guler O., Kahraman N., Kucuk O., Ozpolat B. (2018). Chemopreventive and Antitumor Efficacy of Curcumin in a Spontaneously Developing Hen Ovarian Cancer Model. Cancer Prev. Res. (Phila).

[22] Ireson C., Orr S., Jones D.J., Verschoyle R., Lim C.K., Luo J.L., Howells L., Plummer S., Jukes R., Williams M. (2001). Characterization of metabolites of the chemopreventive agent curcumin in human and rat hepatocytes and in the rat in vivo, and evaluation of their ability to inhibit phorbol ester-induced prostaglandin E2 production. Cancer Res..

[23] Ireson C.R., Jones D.J., Orr S., Coughtrie M.W., Boocock D.J., Williams M.L., Farmer P.B., Steward W.P., Gescher A.J. (2002). Metabolism of the cancer chemopreventive agent curcumin in human and rat intestine. Cancer Epidemiol. Biomark. Prev..

[24] Luthra P.M., Kumar R., Prakash A. (2009). Demethoxycurcumin induces Bcl-2 mediated G2/M arrest and apoptosis in human glioma U87 cells. Biochem. Biophys. Res. Commun..

[25] Yodkeeree S., Chaiwangyen W., Garbisa S., Limtrakul P. (2009). Curcumin, demethoxycurcumin and bisdemethoxycurcumin differentially inhibit cancer cell invasion through the down-regulation of MMPs and uPA. J. Nutr. Biochem..

[26] Hatamipour M., Ramezani M., Tabassi S.A.S., Johnston T.P., Ramezani M., Sahebkar A. (2018). Demethoxycurcumin: A naturally occurring curcumin analogue with antitumor properties. J. Cell Physiol..

[27] Derakhshan A., Chen Z., Van Waes C. (2017). Therapeutic Small Molecules Target Inhibitor of Apoptosis Proteins in Cancers with Deregulation of Extrinsic and Intrinsic Cell Death Pathways. Clin. Cancer Res..

[28] Wang M., Jiang S., Zhou L., Yu F., Ding H., Li P., Zhou M., Wang K. (2019). Potential Mechanisms of Action of Curcumin for Cancer Prevention: Focus on Cellular Signaling Pathways and miRNAs. Int. J. Biol. Sci..

[29] Wong S.Y., Tan M.G., Wong P.T., Herr D.R., Lai M.K. (2016). Andrographolide induces Nrf2 and heme oxygenase 1 in astrocytes by activating p38 MAPK and ERK. J. Neuroinflammation.

[30] Nakashima K., Sato T., Shigemori S., Shimosato T., Shinkai M., Kaneko T. (2018). Regulatory role of heme oxygenase-1 in silica-induced lung injury. Respir. Res..

[31] Hung C.M., Su Y.H., Lin H.Y., Lin J.N., Liu L.C., Ho C.T., Way T.D. (2012). Demethoxycurcumin modulates prostate cancer cell proliferation via AMPK-induced down-regulation of HSP70 and EGFR. J. Agric. Food Chem..

[32] Lin C.Y., Hung C.C., Wang C.C.N., Lin H.Y., Huang S.H., Sheu M.J. (2019). Demethoxycurcumin sensitizes the response of non-small cell lung cancer to cisplatin through downregulation of TP and ERCC1-related pathways. Phytomedicine.

[33] Hatamipour M., Ramezani M., Tabassi S.A.S., Johnston T.P., Sahebkar A. (2019). Demethoxycurcumin: A naturally occurring curcumin analogue for treating non-cancerous diseases. J. Cell Physiol..

[34] Park M., Chae H.D., Yun J., Jung M., Kim Y.S., Kim S.H., Han M.H., Shin D.Y. (2000). Constitutive activation of cyclin B1-associated cdc2 kinase overrides p53-mediated G2-M arrest. Cancer Res..

[35] Ni X., Zhang A., Zhao Z., Shen Y., Wang S. (2012). Demethoxycurcumin inhibits cell proliferation, migration and invasion in prostate cancer cells. Oncol. Rep..

[36] Lal N., Nemaysh V., Luthra P.M. (2018). Proteasome mediated degradation of CDC25C and Cyclin B1 in Demethoxycurcumin treated human glioma U87 MG cells to trigger G2/M cell cycle arrest. Toxicol. Appl. Pharmacol..

[37] Hsiao Y.T., Kuo C.L., Chueh F.S., Liu K.C., Bau D.T., Chung J.G. (2018). Curcuminoids Induce Reactive Oxygen Species and Autophagy to Enhance Apoptosis in Human Oral Cancer Cells. Am. J. Chin. Med..

[38] Chen Y.K., Huse S.S., Lin L.M. (2011). Expression of inhibitor of apoptosis family proteins in human oral squamous cell carcinogenesis. Head Neck.

[39] Tanaka T., Nakayama H., Yoshitake Y., Irie A., Nagata M., Kawahara K., Takamune Y., Yoshida R., Nakagawa Y., Ogi H. (2012). Selective inhibition of nuclear factor-kappaB by nuclear factor-kappaB essential modulator-binding domain peptide suppresses the metastasis of highly metastatic oral squamous cell carcinoma. Cancer Sci..

[40] Almeida L.O., Abrahao A.C., Rosselli-Murai L.K., Giudice F.S., Zagni C., Leopoldino A.M., Squarize C.H., Castilho R.M. (2014). NFkappaB mediates cisplatin resistance through histone modifications in head and neck squamous cell carcinoma (HNSCC). FEBS Open Bio..

[41] Duffey D.C., Crowl-Bancroft C.V., Chen Z., Ondrey F.G., Nejad-Sattari M., Dong G., Van Waes C. (2000). Inhibition of transcription factor nuclear factor-kappaB by a mutant inhibitor-kappaBalpha attenuates resistance of human head and neck squamous cell carcinoma to TNF-alpha caspase-mediated cell death. Br. J. Cancer.

[42] Pae H.O., Jeong G.S., Jeong S.O., Kim H.S., Kim S.A., Kim Y.C., Yoo S.J., Kim H.D., Chung H.T. (2007). Roles of heme oxygenase-1 in curcumin-induced growth inhibition in rat smooth muscle cells. Exp. Mol. Med..

[43] Hsu H.Y., Chu L.C., Hua K.F., Chao L.K. (2008). Heme oxygenase-1 mediates the anti-inflammatory effect of Curcumin within LPS-stimulated human monocytes. J. Cell Physiol..

[44] Lee W.Y., Chen Y.C., Shih C.M., Lin C.M., Cheng C.H., Chen K.C., Lin C.W. (2014). The induction of heme oxygenase-1 suppresses heat shock protein 90 and the proliferation of human breast cancer cells through its byproduct carbon monoxide. Toxicol. Appl. Pharmacol..

[45] Wu M.S., Chien C.C., Chang J., Chen Y.C. (2019). Pro-apoptotic effect of haem oxygenase-1 in human colorectal carcinoma cells via endoplasmic reticular stress. J. Cell Mol. Med..

[46] Wu S.Y., Lee Y.R., Huang C.C., Li Y.Z., Chang Y.S., Yang C.Y., Wu J.D., Liu Y.W. (2012). Curcumin-induced heme oxygenase-1 expression plays a negative role for its anti-cancer effect in bladder cancers. Food Chem. Toxicol..

[47] Ryter S.W. (2019). Heme oxygenase-1/carbon monoxide as modulators of autophagy and inflammation. Arch. Biochem. Biophys..

[48] Almeida A.S., Queiroga C.S., Sousa M.F., Alves P.M., Vieira H.L. (2012). Carbon monoxide modulates apoptosis by reinforcing oxidative metabolism in astrocytes: Role of Bcl-2. J. Biol. Chem..

[49] Jimenez J., Chakraborty I., Mascharak P.K. (2015). Synthesis and assessment of CO-release capacity of manganese carbonyl complexes derived from rigid alpha-diimine ligands of varied complexity. Eur. J. Inorg. Chem..

[50] Lundvig D.M., Pennings S.W., Brouwer K.M., Mtaya-Mlangwa M., Mugonzibwa E., Kuijpers-Jagtman A.M., Wagener F.A., Von den Hoff J.W. (2015). Cytoprotective responses in HaCaT keratinocytes exposed to high doses of curcumin. Exp. Cell Res..

[51] Yang C., Ma X., Wang Z., Zeng X., Hu Z., Ye Z., Shen G. (2017). Curcumin induces apoptosis and protective autophagy in castration-resistant prostate cancer cells through iron chelation. Drug Des. Devel. Ther..

[52] McNally S.J., Harrison E.M., Ross J.A., Garden O.J., Wigmore S.J. (2007). Curcumin induces heme oxygenase 1 through generation of reactive oxygen species, p38 activation and phosphatase inhibition. Int. J. Mol. Med..

[53] Ribeiro F.A., Noguti J., Oshima C.T., Ribeiro D.A. (2014). Effective targeting of the epidermal growth factor receptor (EGFR) for treating oral cancer: A promising approach. Anticancer Res..

[54] Hoch M.A., Cousins K., Nartey R., Riley K., Hartranft M. (2018). Two cases of combination therapy with cetuximab, paclitaxel, and cisplatin for advanced head and neck cancer. J. Oncol. Pharm. Pract..

[55] Guigay J., Even C., Mayache-Badis L., Debbah M., Saada-Bouzid E., Tao Y., Deschamps F., Janot F., Lezghed N., Michel C. (2017). Long-term response in patient with recurrent oropharyngeal carcinoma treated with cetuximab, docetaxel and cisplatin (TPEx) as first-line treatment followed by cetuximab maintenance. Oral Oncol..

[56] Lee J.Y., Lee Y.M., Chang G.C., Yu S.L., Hsieh W.Y., Chen J.J., Chen H.W., Yang P.C. (2011). Curcumin induces EGFR degradation in lung adenocarcinoma and modulates p38 activation in intestine: The versatile adjuvant for gefitinib therapy. PLoS ONE.

[57] Chen C.F., Lu C.C., Chiang J.H., Chiu H.Y., Yang J.S., Lee C.Y., Way T.D., Huang H.J. (2018). Synergistic inhibitory effects of cetuximab and curcumin on human cisplatin-resistant oral cancer CAR cells through intrinsic apoptotic process. Oncol. Lett..

[58] Wada K., Lee J.Y., Hung H.Y., Shi Q., Lin L., Zhao Y., Goto M., Yang P.C., Kuo S.C., Chen H.W. (2015). Novel curcumin analogs to overcome EGFR-TKI lung adenocarcinoma drug resistance and reduce EGFR-TKI-induced GI adverse effects. Bioorg. Med. Chem..

[59] Shao Y., Zhu W., Da J., Xu M., Wang Y., Zhou J., Wang Z. (2017). Bisdemethoxycurcumin in combination with alpha-PD-L1 antibody boosts immune response against bladder cancer. Onco Targets Ther..

[60] Hsieh M.J., Chin M.C., Lin C.C., His Y.T., Lo Y.S., Chuang Y.C., Chen M.K. (2018). Pinostilbene Hydrate Suppresses Human Oral Cancer Cell Metastasis by Downregulation of Matrix Metalloproteinase-2 Through the Mitogen-Activated Protein Kinase Signaling Pathway. Cell Physiol. Biochem..

[61] Chien M.H., Lee W.J., Yang Y.C., Tan P., Pan K.F., Liu Y.C., Tsai H.C., Hsu C.H., Wen Y.C., Hsiao M. (2018). N-alpha-acetyltransferase 10 protein promotes metastasis by stabilizing matrix metalloproteinase-2 protein in human osteosarcomas. Cancer Lett..

