# Supplementary material for: Dual Targeting of the p38 MAPK-HO-1 Axis and cIAP1/XIAP by Demethoxycurcumin Triggers Caspase-Mediated Apoptotic Cell Death in Oral Squamous Cell Carcinoma Cells
Source: Cancers (Basel). 2020 Mar 16;12(3):703. doi: 10.3390/cancers12030703 (PMC7140023; doi:10.3390/cancers12030703)
Supplement: Supplementary file 1 [file cancers-12-00703-s001.pdf]

cancers

Article

Dual targeting of the p38 MAPK-HO-1 axis and cIAP1/XIAP by demethoxycurcumin triggers caspase-mediated apoptotic cell death in oral squamous cell carcinoma cells

Ming-Hsien Chien, Wei-En Yang, Yi-Chieh Yang, Chia-Chi Ku, Wei-Jiunn Lee, Meng-Ying Tsai, Chiao-Wen Lin, Shun-Fa Yang

Supplementary materials and methods

Cell viability assay (MTS assay)

OSCC cells were plated at a density of 3000 cells/well in 96-well plates with complete media and incubated overnight. Cells next received different treatments as indicated for 24 h, and then subjected to a cell-viability assay (MTS assay; Promega, Madison WI) according to the manufacturer's instructions. The absorbance (A) was read at 490 nm using a microplate reader (MQX200; Bio-Tek Instruments, Winooski, VT). Data were collected from three replicates.

Supplementary data

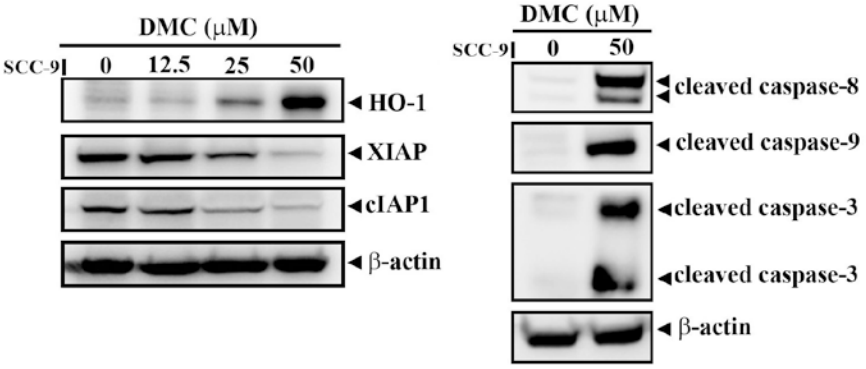

Supplementary Figure S1. Effect of demethoxycurcumin (DMC) on apoptosis-related proteins in oral squamous cell carcinoma (OSCC) cells. SCC-9 cells were treated with indicated concentrations of DMC for 24 h, and a Western blot analysis was used to detect expression levels of heme oxygenase (HO)-1, cellular inhibitor of apoptosis 1 (cIAP1), X-chromosome-linked IAP (XIAP), and cleaved caspase-8/-9/-3. The β-actin protein levels were used to adjust the quantitative results of these protein levels.

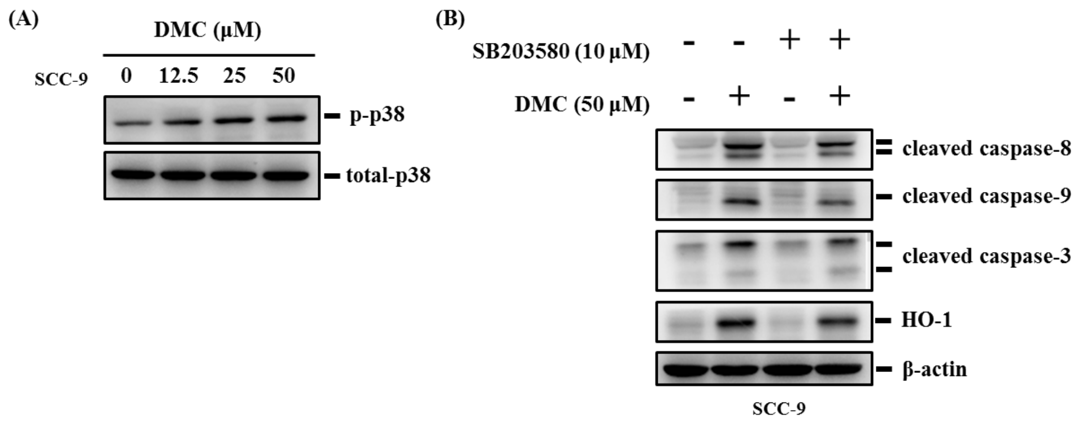

Supplementary Figure S2. Activation of p38 mitogen-activated protein kinase (MAPK) is involved in demethoxycurcumin (DMC)-induced heme oxygenase (HO)-1 expression and cell apoptosis in SCC9 cells. (A) SCC-9 cells were exposed to the vehicle or DMC (12.5~50 μM) for 24 h, then phosphorylation status of p38 MAPK were analyzed by a Western blot analysis. (B) SCC-9 cells were pretreated with SB203580 (10 μM) for 1 h followed by another 24-h vehicle or DMC (50 μM) treatment. Levels of cleaved caspase-3, -8, and -9, and HO-1 were analyzed by a Western blot analysis.

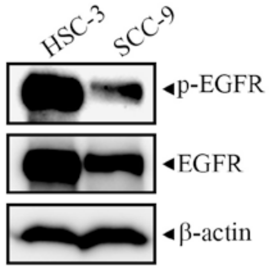

Supplementary Figure S3. Levels of endogenous epidermal growth factor receptor (EGFR) were analyzed by a Western blotting analysis in HSC-3 and SCC-9 oral squamous cell carcinoma (OSCC) cells.

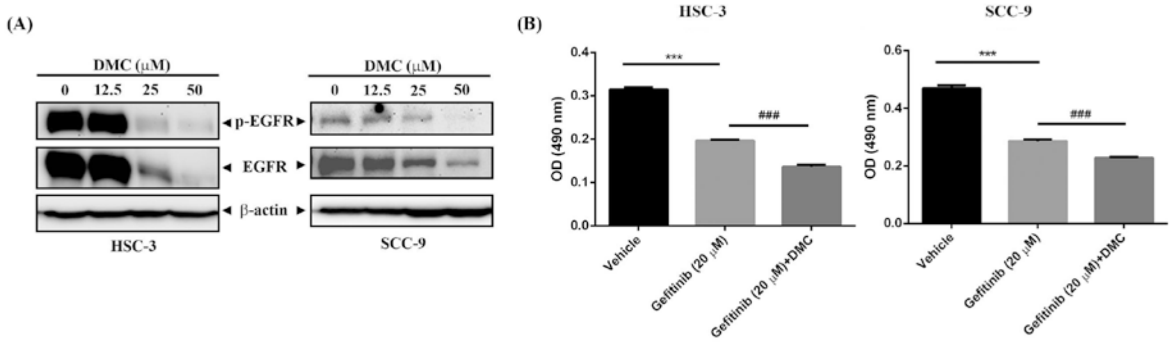

Supplementary Figure S4. Demethoxycurcumin (DMC) potentiates the growth inhibitory effect of gefitinib on oral squamous cell carcinoma (OSCC) cells. (A) SCC-9 and HSC-3 cells were treated with indicated concentrations of DMC for 24 h, and a Western blot analysis was used to detect expression levels of phosphorylated epidermal growth factor receptor (EGFR) and total EGFR. (B) SCC-9 and HSC-3 cells were treated with gefitinib (20 μM) in the presence or absence of the DMC (25 μM) for 24 h, the cell viability was determined by MTS assay. Columns, mean (n=3); bars, SD. ****p* < 0.001 compared with the vehicle group. ###*p* < 0.001 compared with the gefitinib-treated only group.

Whole Blots for Western Blot analysis

Figure 3B

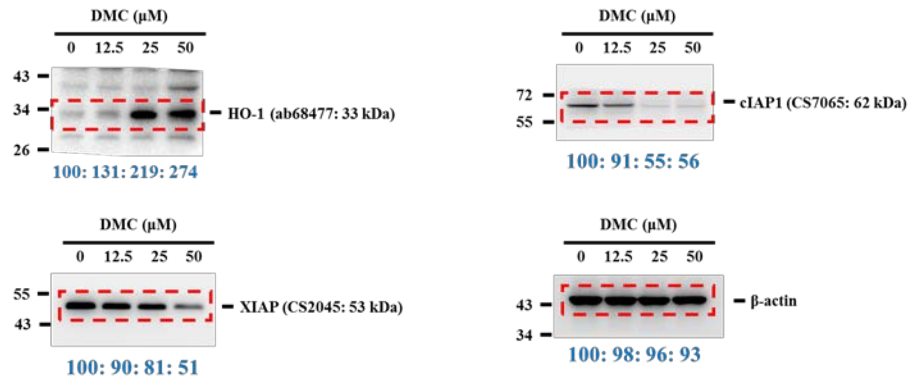

Figure 3D

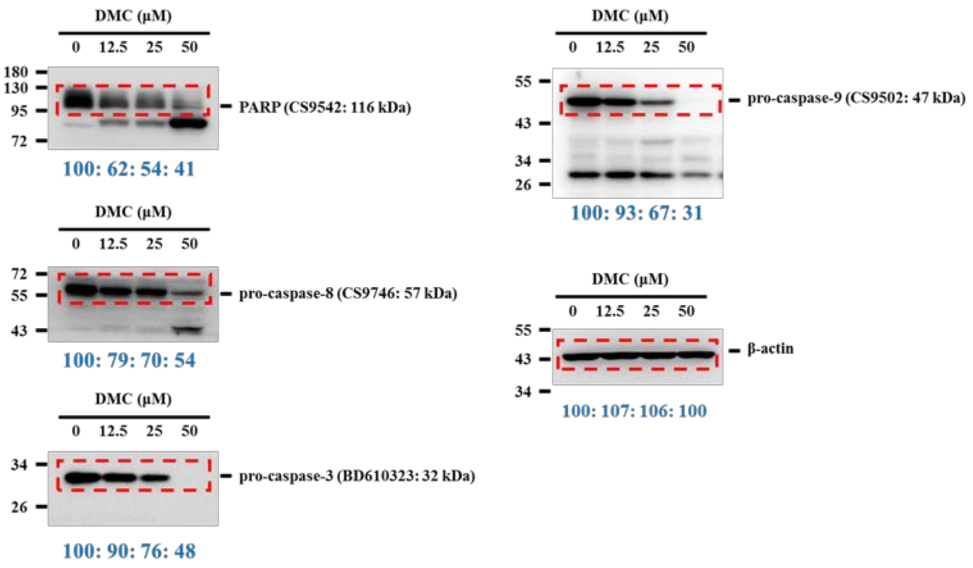

Figure 3F

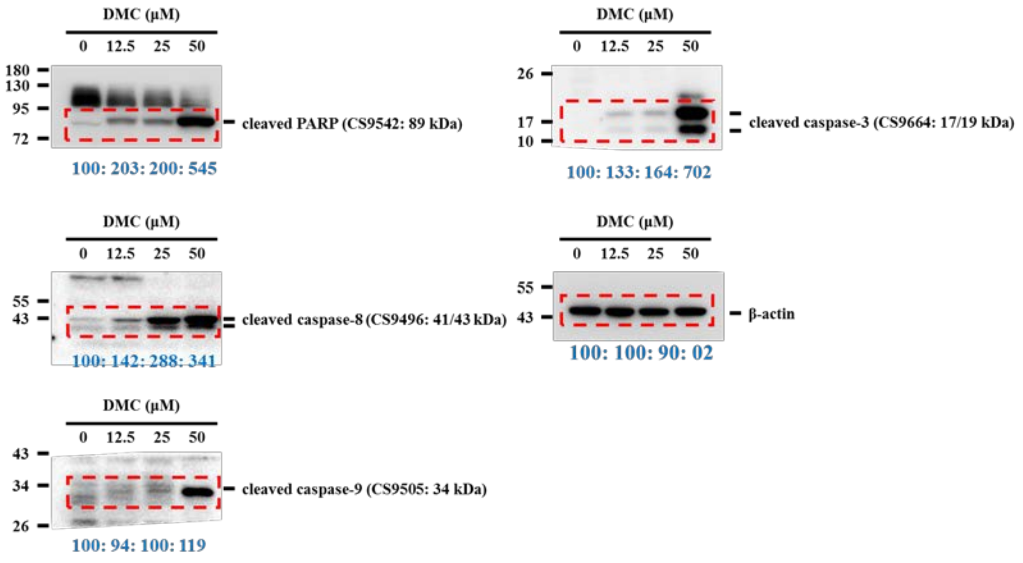

Figure 4A

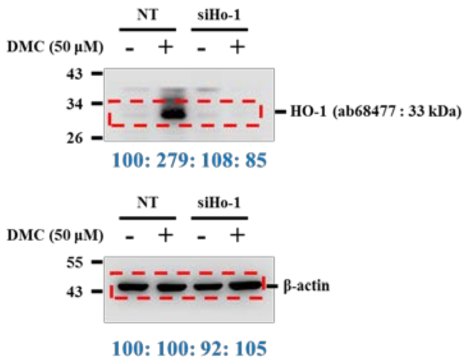

Figure 4B

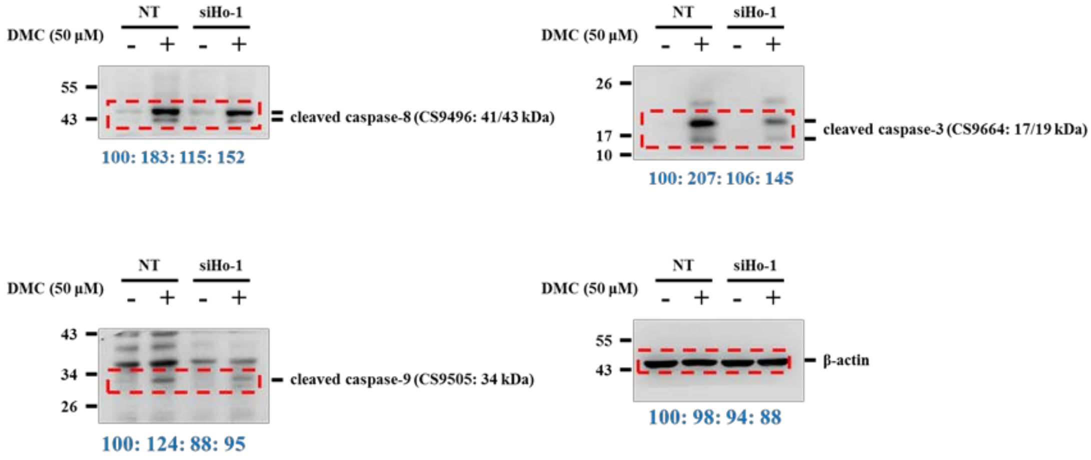

Figure 4E

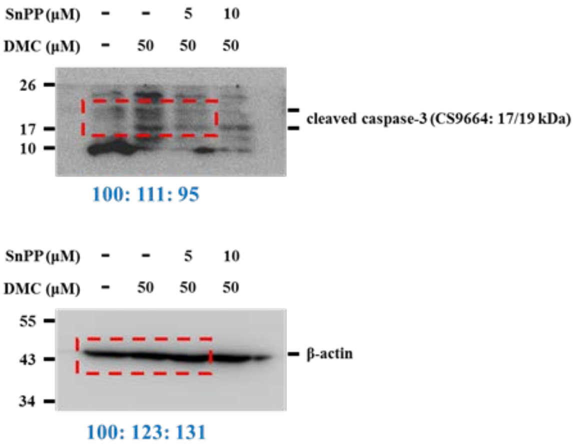

Figure 4I

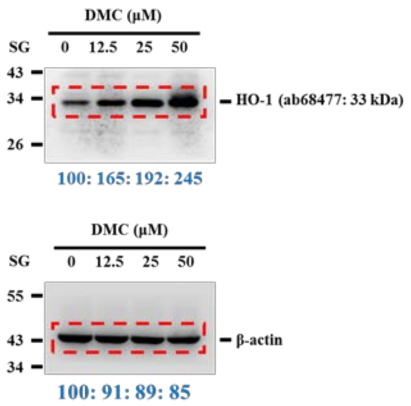

Figure 5A

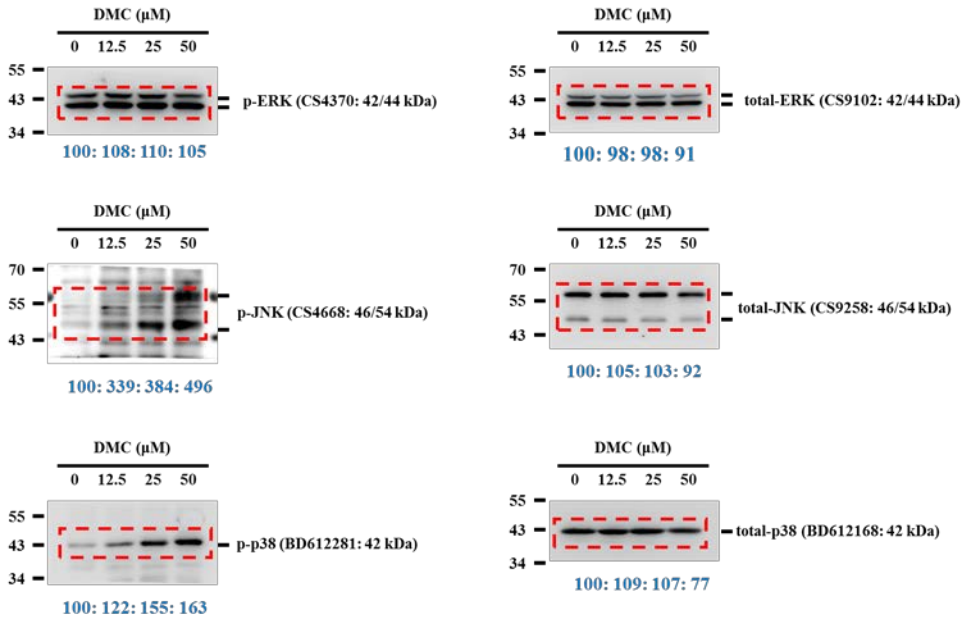

Figure 5C

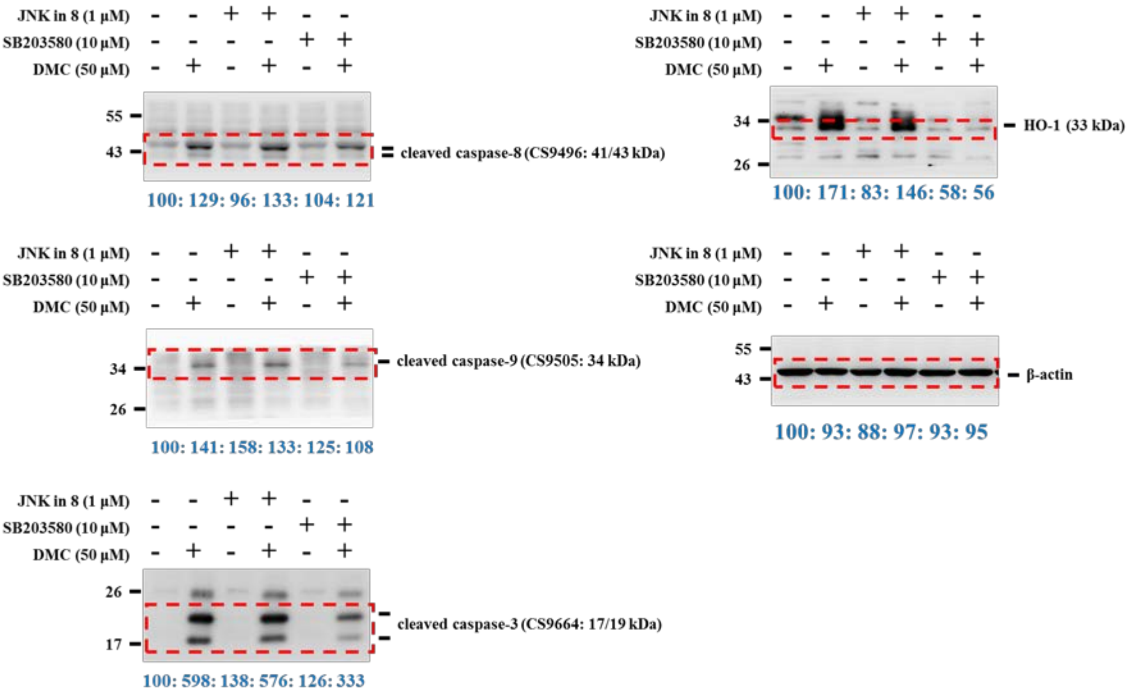

JNK in 8 (1 µM)
SB203580 (10 µM)
DMC (50 µM)
55
43
cleaved caspase-8 (CS9496: 41/43 kDa)
100: 129: 96: 133: 104: 121
34
26
HO-1 (33 kDa)
100: 171: 83: 146: 58: 56
cleaved caspase-9 (CS9505: 34 kDa)
100: 141: 158: 133: 125: 108
β-actin
100: 93: 88: 97: 93: 95
26
17
cleaved caspase-3 (CS9664: 17/19 kDa)
100: 598: 138: 576: 126: 333

Figure S1

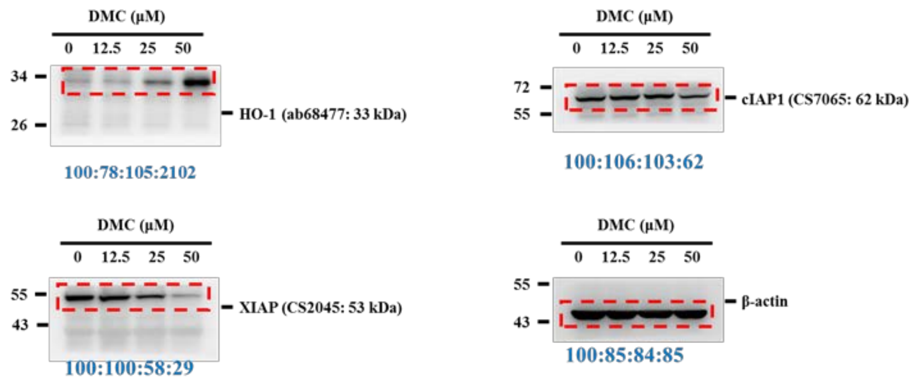

Figure S2A

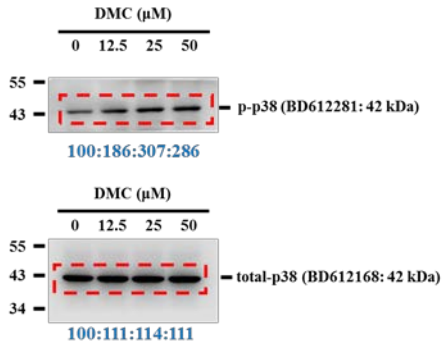

Figure S2B

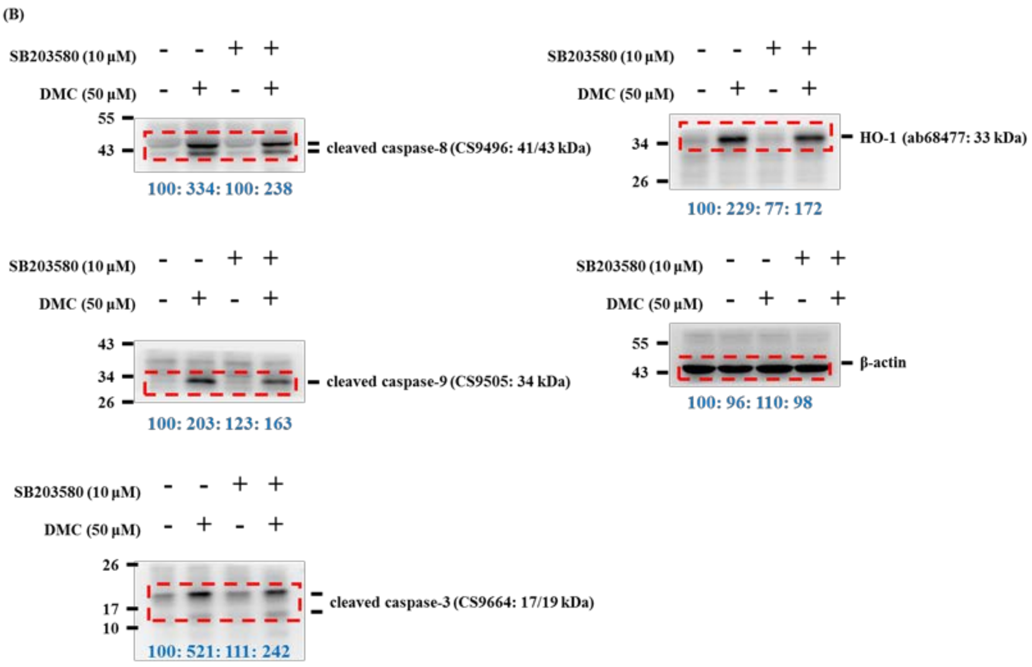

Figure S3

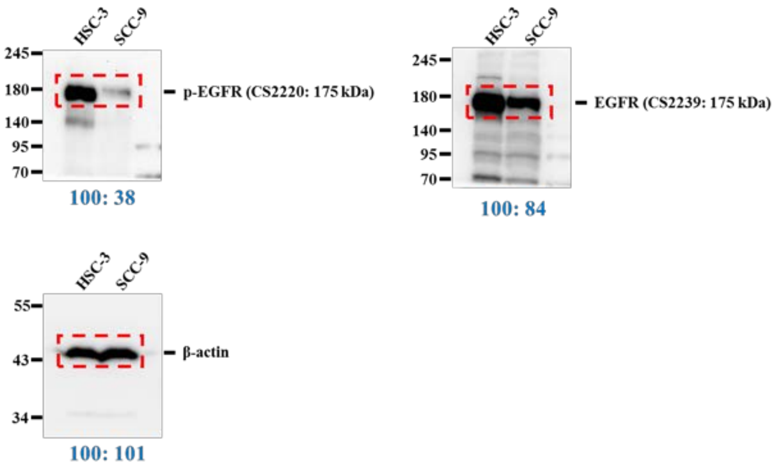

Figure S4A

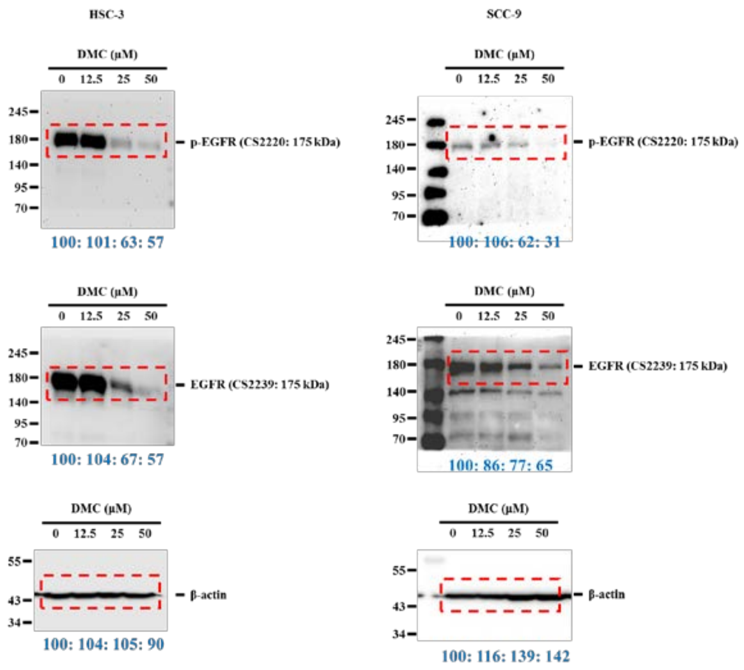

© 2020 by the authors. Licensee MDPI, Basel, Switzerland. This article is an open access article distributed under the terms and conditions of the Creative Commons Attribution (CC BY) license (http://creativecommons.org/licenses/by/4.0/).
